# The Use and Abuse of Tobacco

**Published:** 1859-11

**Authors:** 


ART. VI.—*The Use and Abuse of Tobacco.* By JOHN LIZARS, late Professor of Surgery to the College of Surgery, and lately Senior Operating Surgeon to the Royal Infirmary of Edinburgh. From the eighth Edinburgh edition. 12mo., pp. 128. Philadelphia: Lindsay & Blakiston, 1859.

MILLIONS of dollars are annually spent in the consumption of tobacco. In Great Britain the amount used in 1853 was nearly 30,000,000 pounds, being considerably more, on an average, than one pound to every man, woman, and child in the United Kingdom. In this country, where every male, from the beardless boy to the old man trenching on the verge of the grave, chews and smokes, the consumption of the "weed" must be enormous; and what strikes one as particularly unfortunate is, that so much soil and labor should be wasted in the cultivation of such a villainous plant, which does no other good than to tickle the nose and palate, while the mischief it does is incalculable. No physician or surgeon, now-a-days, uses tobacco as a medicinal agent. The tobacco enema, once so much employed in the treatment of strangulated hernia, has been entirely discarded since the introduction of chloroform and ether. Mr. Macilwain, of London, is perhaps almost the only respectable surgeon who still resorts to the practice.

Of all the forms in which tobacco is used that of smoking is undoubtedly the least offensive, but even that is sufficiently disgusting. Napoleon said it was a habit only fit to amuse sluggards. It is difficult to conceive how a confirmed chewer can be tolerated by a delicate and refined wife. His filthy mouth and horrid breath must be an abomination to her. Then, look at the nasty nose of the man who habitually takes snuff; see how he frets his nasal proboscis, how he blunts the sense of smell, and how he excites the flow of mucus. Oh, what a beast! What a sad perversion of man's noble organs! But so it is. We may write, and talk, and scold about the use of tobacco as long as we please, and still chewing, and smoking, and snuffing will go on at the same pace as ever—perhaps, indeed, all the faster for our opposition: such is the world's stubbornness. If we were an autocrat we should send every one to the mines that indulges in these filthy practices, and confiscate his estate in the bargain; nay, more, we should deny him the benefit of clergy in his dying moments. We should eradicate the use of the poisonous weed at all hazard, even at the risk of exterminating a goodly portion of the human race. Why should we hesitate? for, if Mr. Lizars and other writers, who have so profoundly investigated this matter, are to be believed, a rapid degeneration is going on, affecting every part of man's body, and threatening to reduce him to the most abject physical and mental condition. One of the most degrading of these effects is a diminution of stature, a process which, in time, may—*mirabile dictu!*—change us to the condition of Lilliputians, and thus wickedly thwart God's providence in regard to the world's destiny. The contemplation of such a scene is most painful, and yet it is precisely what, if we implicitly believe these writers, may ultimately occur.

While we fully credit all that Mr. Lizars so earnestly inculcates in this little brochure concerning the degenerating influences of tobacco upon man's stature and mental powers, we are obliged to dissent from him in regard to other subjects, which, although well argued, are not so well sustained by sound logical deductions. We allude more particularly to the idea that smoking and chewing are a frequent cause of cancer of the tongue, lip, and gums. We have seen many cases of this disease in these situations, and not a few in persons that had never employed tobacco in any form whatever. We are sure that Mr. Lizars is not borne out in his conclusions in his statements of this subject. The concurrence of carcinoma of these parts and of the use of this nasty weed is sufficiently common, but it does not thence follow that they should be viewed in the light of cause and effect. A careful study of the subject has satisfied us that the development of the disease may, under such circumstances, be readily explained by the laws of coincidence. Our author has some very curious notions about this matter. We quote the following paragraphs in illustration of our position:—

"From the cases I have recorded, I may presume that a person with a cancerous diathesis, or predisposition or constitution, smoking a cutty pipe, must be liable to communicate the disease to another who might take up the same pipe.

"In the syphilitic constitution, the mucous membrane of the mouth is very prone to excitement and ulceration; and if the latter is produced by smoking tobacco, the ulceration, in nine cases out of ten, will degenerate into cancerous or cancroid ulceration, and prove fatal, after lingering and cruel sufferings."

Surely our learned author cannot be in earnest when he makes such statements; such assertions may do very well to frighten persons who smoke and chew, but there is no pathologist, of the least sense or experience, who would accord to them his approval. Really, Mr. Lizars, this will not do; we hate and detest tobacco in all its forms, except when it is used for killing vermin; but we cannot, in any manner in which we can "fix" it, indorse the far-fetched and unphilosophical conclusions arrived at in the above quotations. If the "cutty pipe" can perform such wonders, we confess that we have never, in a practice of no mean extent, witnessed any proof of it.

Mr. Lizars, while on the subject of cancer of the tongue, indulges in some conjectures regarding the minute structure of that organ, and enters his caveat against its removal when affected with malignant disease. He refers to two cases in which the tongue was excised by Mr. Syme, both of which terminated fatally, and he evidently introduces the notice in order that he may give that gentleman a thrust under the rib. One would have supposed, he remarks, that such a result would have made any surgeon acquainted with pathology pause before excising such a vascular organ lying so near the lungs. But, as John Bell says, operations have come to represent at last the whole science. Such seems to be the case at least in the opinion of the so-called "First Surgeon in Europe."

Mr. Lizars and Mr. Syme evidently dislike each other, and so our tobacco warrior seizes the occasion, dragged in, *vi et armis*, to spit a little of the juice of the weed into the face of the so-called "First Surgeon in Europe." Edinburgh has long been famous for its medical quarrels.

We close our notice of this little treatise with no great prepossessions in its favor. Mr. Lizars has collected many facts of interest, but we are far from thinking that he has made the best use of them. Nevertheless, the work is very readable, and we therefore bespeak for it an attentive perusal.

